# ALS-linked TDP-43^M337V^ knock-in mice exhibit splicing deregulation without neurodegeneration

**DOI:** 10.1186/s13041-020-0550-4

**Published:** 2020-01-20

**Authors:** Seiji Watanabe, Kotaro Oiwa, Yuri Murata, Okiru Komine, Akira Sobue, Fumito Endo, Eiki Takahashi, Koji Yamanaka

**Affiliations:** 1 Department of Neuroscience and Pathobiology, Research Institute of Environmental Medicine, Nagoya University, Nagoya, Aichi 464-8601 Japan; 2 Department of Neuroscience and Pathobiology, Nagoya University Graduate School of Medicine, Nagoya, Aichi 466-8550 Japan; 3 Department of Neurology, Nagoya University Graduate School of Medicine, Nagoya University, Nagoya, Aichi 466-8550 Japan; 4 Support Unit for Animal Resources Development, Research Resource Division, RIKEN Center for Brain Science, Wako, Saitama, 351-0198 Japan

**Keywords:** Amyotrophic lateral sclerosis (ALS), TDP-43, TDP-43 knock-in mice

## Abstract

Abnormal accumulation of TAR DNA-binding protein 43 (TDP-43), a DNA/RNA binding protein, is a pathological signature of amyotrophic lateral sclerosis (ALS). Missense mutations in the *TARDBP* gene are also found in inherited and sporadic ALS, indicating that dysfunction in TDP-43 is causative for ALS. To model TDP-43-linked ALS in rodents, we generated TDP-43 knock-in mice with inherited ALS patient-derived TDP-43^M337V^ mutation. Homozygous TDP-43^M337V^ mice developed normally without exhibiting detectable motor dysfunction and neurodegeneration. However, splicing of mRNAs regulated by TDP-43 was deregulated in the spinal cords of TDP-43^M337V^ mice. Together with the recently reported TDP-43 knock-in mice with ALS-linked mutations, our finding indicates that ALS patient-derived mutations in the *TARDBP* gene at a carboxyl-terminal domain of TDP-43 may cause a gain of splicing function by TDP-43, however, were insufficient to induce robust neurodegeneration in mice.

## Main text

Abnormal accumulation of TDP-43 has been identified as a pathological signature of amyotrophic lateral sclerosis (ALS), an adult neurodegenerative disease characterized by a selective loss of motor neurons, and a part of frontotemporal dementia (FTD) [1]. Cytoplasmic accumulation of TDP-43 with a loss of TDP-43 from nuclei, known as a TDP-43 pathology, is observed in almost all forms of ALS, including sporadic and familial ALS. To date, more than 50 mutations in the *TARDBP* gene, encoding TDP-43, have been identified in inherited and sporadic ALS, implicating TDP-43 dysfunction as a central component for ALS pathogenesis [2]. TDP-43 is a ubiquitously expressed DNA/RNA binding nuclear protein and plays multifunctional roles in RNA metabolism, including pre-mRNA splicing, translational control, and mRNA stability [3]. Of note, TDP-43 is known to control its own mRNA stability through binding to the 3′ UTR, indicating that the level of TDP-43 protein is tightly regulated [3]. Indeed, overexpression of wild-type TDP-43 in mice induces neurodegeneration, whereas elimination of TDP-43 leads to embryonic lethality [4, 5]. However, it is still unclear whether dysfunction in TDP-43 leads to neurodegeneration through a gain or loss of TDP-43 function. To model TDP-43-mediated neurodegeneration in mice, several lines of transgenic mice have been developed and reproduced some features of neurodegeneration observed in human ALS/FTD. However, the overexpression approach has a limitation in differentiating the role between wild-type and mutant TDP-43 in motor neuron health and disease in mice [4, 5].

**Fig. 1:**
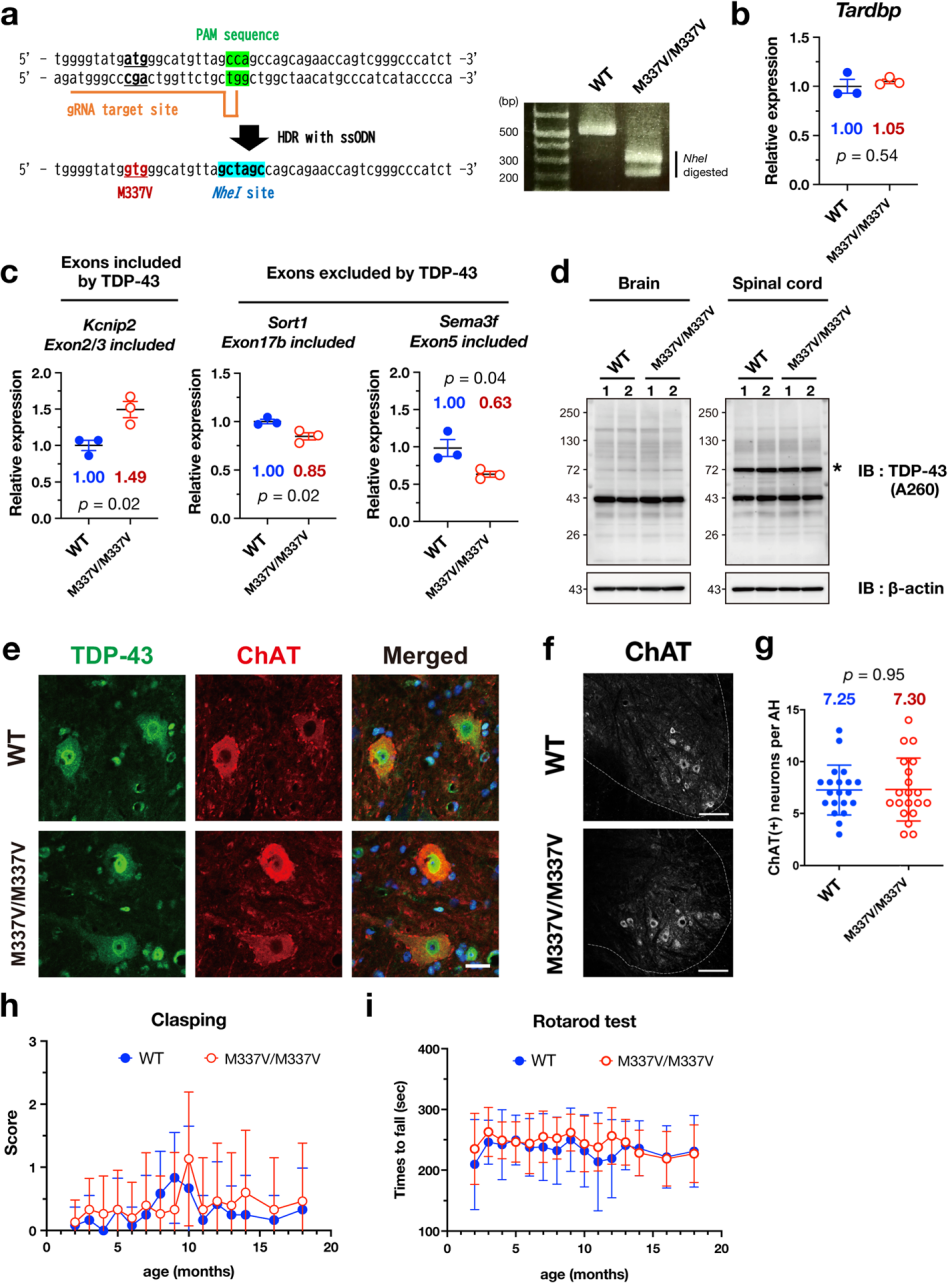
Characterization of TDP-43^M337V^ knock-in mice. **a** Schematic illustration of introducing TDP-43^M337V^ mutation into an endogenous murine *Tardbp* exon 6 (left panel). The representative genotyping result is also shown (right panel). *Nhe I* restriction site is introduced in the mutant allele, resulting in no change of the amino acid at *Nhe I* site. **b** The expression level of *Tardbp* mRNA was not altered in the brains of 700-days-old TDP-43^M337V/M337V^ (M337 V/M337 V) mice and wild-type (WT) littermates. **c** Alternation in splicing of genes regulated by TDP-43. The level of mRNA containing exons included by TDP-43 (*Kcnip2* exon 2 and 3) was increased, while the levels of mRNA containing exons excluded by TDP-43 (*Sort1* exon 17b and *Sema3f* exon 5) were reduced, suggesting a gain-of-function mechanism in TDP-43^M337V/M337V^ mice. Relative expression levels of mRNA normalized to the WT control are plotted with SD (*n* = 3 each (**b**, **c**)) and were analyzed by unpaired *t*-tests. **d** Representative immunoblots of TDP-43 and β-actin in the brains and spinal cords of 700-days-old TDP-43^M337V/M337V^ mice and WT littermates. Asterisk denotes a non-specific band. **e** and **f** Representative immunofluorescence images of the anterior horn in lumbar spinal cords of 700-days-old TDP-43^M337V/M337V^ mice and WT littermates stained with anti-TDP-43 (3H8, green) and anti-ChAT (red) antibodies along with the merged images. TDP-43 was not mislocalized in motor neurons of TDP-43^M337V/M337V^ mice (**e**). Low magnification images stained with anti-ChAT antibody (**f**). Scale bars: 20 μm (**e**), 100 μm (**f**). **g** Quantification of the numbers of ChAT-positive motor neurons per each anterior horn (AH) in the lumbar spinal cords of 700-days-old TDP-43^M337V/M337V^ mice and WT littermates. For quantification, 20 AHs in three animals per each genotype were counted, and data are plotted as mean ± SD, and were analyzed by an unpaired *t*-test. **h** and **i** TDP-43^M337V/M337V^ mice did not show any motor dysfunction phenotypes in the measurement of clasping score (**h**) and rotarod test (**i**). Data are plotted as mean ± SD, and were analyzed with two-way ANOVA. *n* = 15 for WT and 14 for M337 V/M337 V

Based on these backgrounds, we set out to create a knock-in mouse model carrying an ALS patient-derived mutation in the murine *Tardbp* gene. Of more than 50 known mutations, we chose TDP-43^M337V^ mutation for the following reasons: TDP-43^M337V^ protein has a long half-life in cells, the ALS patients with this mutation show earlier disease onset [6, 7], and an amino acid sequence of 241–414 including a methionine residue at position 337 is highly conserved among mouse and human. We engineered mice with n.1009 A > G (M337V) mutation in the murine *Tardbp* gene by utilizing CRISPR/Cas9 genome-editing technology (Additional file 1). Both homozygous and heterozygous mice carrying the allele of TDP-43^M337V^ developed normally as recently reported (Fig. 1a, Additional file 1: Figure S1, S2) [8].

TDP-43 plays a pivotal role in regulating alternative splicing as well as controlling the level of TDP-43 mRNA itself by a negative feedback mechanism. Therefore, we first examined whether ALS-linked TDP-43^M337V^ mutation affects the expression level of its own mRNA in mice. Analysis of TDP-43 mRNA levels in the brains of 700-days-old homozygous TDP-43^M337V^ mice (TDP-43^M337V/M337V^) revealed that there was no difference in expression level between wild-type and TDP-43^M337V/M337V^ mice (Fig. 1b). In addition, the mRNA levels of *Notch1* and *Nek1*, known as TDP-43 target genes, were unaffected by homozygous M337 V mutation (Additional file 1: Figure S3). We next examined whether TDP-43^M337V^ deregulates alternative splicing of mRNAs that are known as splicing targets of TDP-43. Among the several splicing targets examined, we found a 1.49-fold increase in inclusion of *Kcinp2* exon 2/3, a 0.85-fold decrease in exclusion of *Sort1* exon 17b, and a 0.63-fold decrease in exclusion of *Sema3f* exon 5 in the brain of TDP-43^M337V/M337V^ mice (Fig. 1c). Although there were no significant changes in other splicing targets, *Poldip3* and *Eif4h* (Additional file 1: Figure S4), changes in splicing of *Kcinp2, Sort1, and Sema3f* in TDP-43^M337V/M337V^ mice are consistent with a gain of TDP-43 function [9, 10].

Since the mislocalization of TDP-43 protein in cytoplasm is a pathological signature of ALS, we examined subcellular localization of TDP-43^M337V^ mutant protein in the affected tissue in TDP-43^M337V/M337V^ mice. Both mutant and wild-type TDP-43 proteins expressed at the similar level, and were predominantly localized in nucleus of brain and spinal cords of 700-days-old TDP-43^M337V/M337V^ and wild-type mice (Fig. 1d, e), suggesting that disease-causing missense mutation in TDP-43 alone did not alter the protein level itself and was insufficient to induce protein mislocalization in mice. Moreover, carboxyl-terminal (C-terminal) fragments of TDP-43, characteristic of TDP-43 pathology, were not detected in the brains and spinal cords of TDP-43^M337V/M337V^ mice (Fig. 1d), and there was no detectable loss of motor neurons or reactive gliosis in TDP-43^M337V/M337V^ mice (Fig. 1e-g, Additional file 1: Figure S5). Nuclear Gems, where SMN complex resides to control splicing, are known to be regulated by TDP-43 and FUS [11–13]. In ventral horn neurons of TDP-43^M337V/M337V^ mice, the number of nuclear Gems was not altered (Additional file 1: Figure S6). We further examined whether TDP-43^M337V/M337V^ mice show motor dysfunction with aging. Measurement of rotarod and clasping scores as well as body weights revealed no difference in those scores between TDP-43^M337V/M337V^ and wild-type mice until 18 months old (Fig. 1h, i, Additional file 1: Figure S2).

The present study demonstrates that homozygous TDP-43^M337V^ mice generated by CRISPR/Cas9 show splicing deregulation of some TDP-43 target mRNAs without apparent deterioration in motor function and pathology until 20 months old. Recently, homozygous TDP-43^Q331K^ knock-in mice showed a reduced number of parvalbumin-positive interneurons and cognitive dysfunction with phenotypic heterogeneity [9]. Homozygous TDP-43^G298S^ or TDP-43^M337V^ knock-in mice showed very mild denervation of hindlimbs at 2.5 years of age [8]. Besides, heterozygous TDP-43^M323K^ mice, generated by *N*-ethyl-*N*-nitrosourea (ENU) random mutagenesis, showed modest neurodegenerative phenotype [10]. These mutant mice uniformly show very mild phenotypes, likely because the 2-years-life span of rodents may be insufficient to induce neurodegeneration derived from splicing deregulation caused by mutant TDP-43.

Our TDP-43^M337V/M337V^ mice showed splicing deregulation of TDP-43 target mRNAs, *Kcinp2, Sort1, and Sema3f*, suggesting that M337 V mutation causes a gain of function in TDP-43. Gain of TDP-43 function is also suggested in TDP-43^Q331K^ and TDP-43^M323K^ mice [9, 10]. All three missense mutations discussed here are located in the low complexity region at the C-terminal of TDP-43, suggesting that ALS-causing TDP-43 mutations in the C-terminal region may cause gain of TDP-43 function, at least, at an initial disease stage. This point makes a good contrast with the role of N-terminal TDP-43 fragment in dominant-negative function in TDP-43 [14]. In our study, the mRNA and protein levels of TDP-43 were unchanged in TDP-43^M337V^ mice, while they were moderately upregulated in TDP-43^Q331K^ [9]. This difference may explain the more modest phenotype of our TDP-43^M337V^ mice.

All knock-in mice carrying ALS-linked missense mutations in TDP-43 do not show robust TDP-43 pathology even in homozygous mutant mice. Perhaps, additional conformational change of TDP-43 protein may be needed to develop TDP-43 pathology. Finally, our results from TDP-43^M337V^ knock-in mice further strengthen the findings that mutations at the C-terminal region of TDP-43 likely cause a gain of TDP-43 splicing function at an initial stage of the disease, which may be followed by the loss of TDP-43 function due to a loss of TDP-43 proteins from nuclei.

## Supplementary information

**Additional file 1. **Material and methods. **Figure S1.** Direct sequencing of *Tardbp* gene exon 6 in heterozygous TDP-43^M337V^ knock-in mice. **Figure S2.** Body weights were not affected in TDP-43^M337V^ knock-in mice. **Figure S3.** Relative expression levels of *Notch1* and *Nek1* mRNAs were not altered in the brain of aged homozygous TDP-43^M337V^ mice. **Figure S4.** Splicing was not altered in *Eif4h* or *Poldip3*, which are also regulated by TDP-43, in the brain of aged homozygous TDP-43^M337V^ mouse brains. **Figure S5.** Gliosis was not observed in ventral horn of aged (700 days-old) homozygous TDP-43^M337V^ mice. **Figure S6.** The number of Gems was not affected in ventral horn neurons of aged (700 days-old) homozygous TDP-43^M337V^ mice.

## Data Availability

The datasets used and/or analyzed during the current study are available from the corresponding author on reasonable request.

## Abbreviations

ALS: Amyotrophic Lateral Sclerosis
ChAT: Choline acetyltransferase
FTD: Frontotemporal dementia
SMN: Survival of motor neurons
TDP-43: TAR DNA-binding protein 43
