# Supplementary material for: ALS-linked TDP-43M337V knock-in mice exhibit splicing deregulation without neurodegeneration
Source: Mol Brain. 2020 Jan 20;13:8. doi: 10.1186/s13041-020-0550-4 (PMC6971932; doi:10.1186/s13041-020-0550-4)
Supplement: Supplementary file 1 — Additional file 1. Material and methods. Figure S1. Direct sequencing of Tardbp gene exon 6 in heterozygous TDP-43M337V knock-in mice. Figure S2. Body weights were not affected in TDP-43M337V knock-in mice. Figure S3. Relative expression levels of Notch1 and Nek1 mRNAs were not altered in the brain of aged homozygous TDP-43M337V mice. Figure S4. Splicing was not altered in Eif4h or Poldip3, which are also regulated by TDP-43, in the brain of aged homozygous TDP-43M337V mouse brains. Figure S5. Gliosis was not observed in ventral horn of aged (700 days-old) homozygous TDP-43M337V mice. Figure S6. The number of Gems was not affected in ventral horn neurons of aged (700 days-old) homozygous TDP-43M337V mice. [file 13041_2020_550_MOESM1_ESM.pdf]

Additional File 1

ALS-linked TDP-43^{M337V} knock-in mice exhibit splicing deregulation without neurodegeneration

Seiji Watanabe, Kotaro Oiwa, Yuri Murata, Okiru Komine, Akira Sobue, Fumito Endo, Eiki Takahashi, and Koji Yamanaka

1. Material and methods

Animals

crRNA (5'-GCCCGACUGGUUCUGCUGGCGUUUUAGAGCUAUGCUGUUUUG-3'), trancRNA, and single-strand oligodeoxynucleotide (ssODN; 5'-CTTTTAGCATTAACCCAGCGATGATGGCTGCGGCTCAGGCAGCGTTGCAGAGCAGTTGGGGTATGGTGGGCATGTTAGCTAGCCAGCAGAACCAGTCGGGCCCATCTGGGAATAACCAAAGCCAGGGCAGCATGCAGAGGGAACCAAAT-3') for homologous directed repair (HDR) were synthesized by FASMAC Co., Ltd. (Kanagawa, Japan). The mixture of crRNA, trancrRNA, ssODN, and recombinant Cas9 protein (Thermo Fisher Scientific Inc., Waltham, MA, USA) were prepared as described in previous studies [1, 2]. All the components above were mixed in 50 µL Tris-EDTA buffer (1 mM Tris-HCl, 10 µM ethylenediaminetetraacetic acid (EDTA), pH 8.0). The mixture was incubated at 37 ºC for 15 min, then injected into pronuclei of one-cell-stage zygotes of C57BL/6J mice. The correctly edited animals were confirmed by direct sequencing of *Tardbp* gene exon 6. The animals were maintained in the C57BL/6J genetic background. Genotyping of the mice was performed using nested PCR. Briefly, *Tardbp* gene exon 6 was amplified using the following primers: 5'-TTTGCCTTCGTCACCTTTGC -3' and 5'-CACAGCCTTGCGTTCATAGC-3. The first-round amplicons were used as templates for second round PCR with primers below: 5'-GTTGCCCAGTCTCTTTGTGGAGAGG-3' and 5'-TCCCCAGCCAGAAGACTTAGAATCC-3'. Then, 250 ng of the second-round amplicons were digested with *Nhe I* enzyme (Takara Bio, Shiga, Japan) to detect the edited alleles. The mice were housed in the specific pathogen-free (SPF) environment (12 h light-dark-cycle; 23 ± 1 ºC; 50 ± 5 % humidity), and treated in compliance with the requirements of the Animal Care and Use Committee, Nagoya University and RIKEN.

Hindlimb clasping and Rotarod test

Hindlimb clasping was scored from 0 (normal) to 3 (entirely retracted) in 10 seconds observation as described in a previous study [3]. Rotarod tests were performed as previously reported [4]. In brief, the mice were placed on the rotating rods, which accelerated from 0 to 30 rpm for 5 minutes with 15 min interval among each trial (Muromachi Kikai, Tokyo Japan). The most prolonged latencies to fall off the rotating rods out of three trials were scored. No randomization or blinding was used in this study.

Antibodies

Following primary antibodies were used in this study: anti-choline acetyl transferase (ChAT) (1:100, #AB144P, Merck Millipore Corp., Billerica, MA, USA), anti-TDP-43(3H8) (1:2000 for immunofluorescence, 1:1,000 for immunoblotting, #MABN45, Merck Millipore), anti-TDP-43(A260) (1:1000, #3449, RRID:AB_2200511, Cell Signaling), anti-TDP-43 (1:500, #10782-2-AP, RRID: AB_615042, Proteintech group Inc., Chicago, IL, USA), anti-β-actin (1:5000, #A5441, RRID: AB_476744, Sigma-Aldrich), anti-glial fibrillary acidic protein (GFAP) (1:250, #G3893, RRID: AB_477010, Sigma-Aldrich), anti-Iba1 (1:500, #019-19741, RRID: AB_839504, Wako Pure Chemical Industries Ltd., Osaka, Japan), and anti-SMN (1:1,000, #610646, RRID: AB_397973, BD Biosciences, San Jose, CA, USA).

Immunofluorescence

Immunofluorescence analyses were performed as described elsewhere [4]. Briefly, tissues were fixed with 4 %(w/v) paraformaldehyde in 0.1 M phosphate buffer, embedded in Tissue-Tek OCT compound medium (Sakura Finetek, Tokyo, Japan), and frozen at −80 ºC until use. After blocking, the 15 μm-sliced spinal cord sections were incubated with primary antibodies overnight at 4°C. Bound primary antibodies were detected with Alexa Fluor 488- or Alexa Fluor 546-conjugated secondary antibodies (all used in 1:1000) (Thermo Fisher). Immunofluorescence images were obtained by a confocal laser scanning microscopy (LSM-700; Carl Zeiss AG, Oberkochen, Germany) and the equipped software (Zen; Carl Zeiss AG).

Immunoblotting

Immunoblotting analyses were performed as described elsewhere [4]. Total protein concentration was measured using the Bio-rad protein assay kit as described in the manufacturer's instructions (Bio-rad, Hercules, CA, USA). Aliquots of 20 μg proteins were analyzed by immunoblotting. The membranes were visualized with Immobilon Crescendo Western HRP substrate (#WBLUR0100, Merck Millipore) according to the manufacturer's protocol after

incubation with horseradish peroxidase (HRP)-conjugated anti-rabbit (1:5000, #NA934, RRID: AB_772206, GE Healthcare, Waukesha, WI, USA) or anti-mouse (1:5000, #NA931, RRID: AB_772210, GE Healthcare) secondary antibodies. Densitometric analyses were performed by using an image analyzer LAS-4000 mini (Fuji-film, Tokyo, Japan) with the equipped software (Multi Gauge; Fuji-film).

RNA isolation and quantitative reverse transcription (RT)-PCR

Total RNA isolation from mouse cerebral cortex and quantitative RT-PCR were performed as described previously [4]. Relative mRNA expression was calculated by a standard curve method normalized to β-actin gene (Actb) and relative to the control samples. All samples were run in duplicate. The primers that were used in this study are designed with GetPrime [5] or in the previous study [4, 6], and listed as follows: for *Tardbp*; 5′-AAAAGGAAAATGGATGAGACAGATG-3′ and 5′-AACTGAGCAGGATCTGAAAGACTATTT-3′, for *Notch1*; 5’-TGGATGACCTAGGCAAGTC-3’ and 5’- TTCTGCATGTCCTTGTTGG-3’, for *Nek1*; 5’-AACATGCATTTGAAGCTGGA-3’ and 5’-AATGTGGAGACACTGGAGG-3’, for *Sort1* including exon 17b; 5’-AACCCCACAAAGCAGGACT-3’ and 5’-CTGCTACGACTGTGACAAGC-3’, for *Kcnip2* including exon 2 and 3; 5’-CAGTGAAACATTAGCTGCC-3’ and 5’-TTCAAACTCATCCTCCACG-3’, for *Sema3f* including exon 5; 5’-ATGTGCACCTATGTGAACC-3’ and 5’-AAGATGTAATCCTGGCGTG-3’, for *Poldip3* including exon 3; 5’-GGCTAAACAGAACTTGTATGACC-3’ and 5’-GCTGCAAACTTCATCTGCT-3’, for Eif4h including exon 5; 5’-GGTGGACCTGATGACAGAG-3’ and 5’- GTCATCCCTGTAGCCAGAG-3’, for quantifying mRNA levels of β-actin; 5′-GCTATGTTGCTCTAGACTTCG-3′ and 5′-GGATTCCATACCCAAGAAGG-3′.

Statistics

Time-courses of clasping and rotarod scores were analyzed by two-way ANOVA and unpaired t-test. All the data from immunofluorescence, semi-quantitative immunoblotting, and quantitative RT-PCR were analyzed by t-test for comparison between 2 groups, or one-way ANOVA followed by the post-hoc Tukey’s multiple comparison t-test for comparison among more than 3 groups, respectively. All the statistical analyses were carried out using GraphPad Prism software (GraphPad Software, La Jolla, CA).

2. Additional Figures

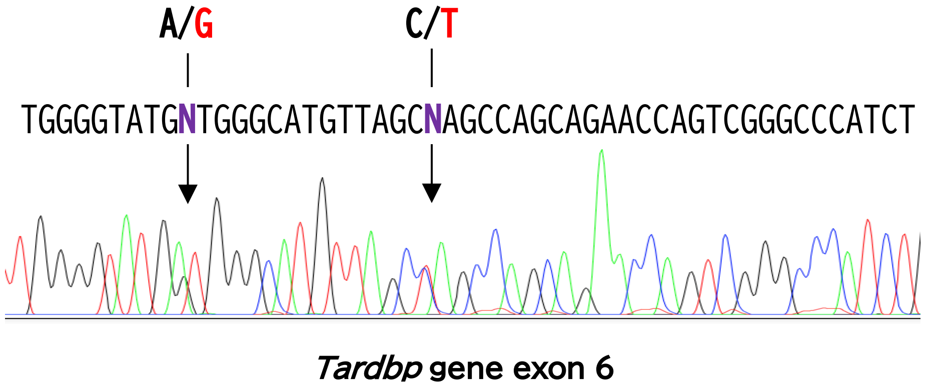

Figure S1. Direct sequencing of *Tardbp* gene exon 6 in heterozygous TDP-43^{M337V} knock-in mice

Representative electropherogram of *Tardbp* gene exon 6 in heterozygous TDP-43^{M337V} knock-in mice indicates that the desired mutations are correctly introduced.

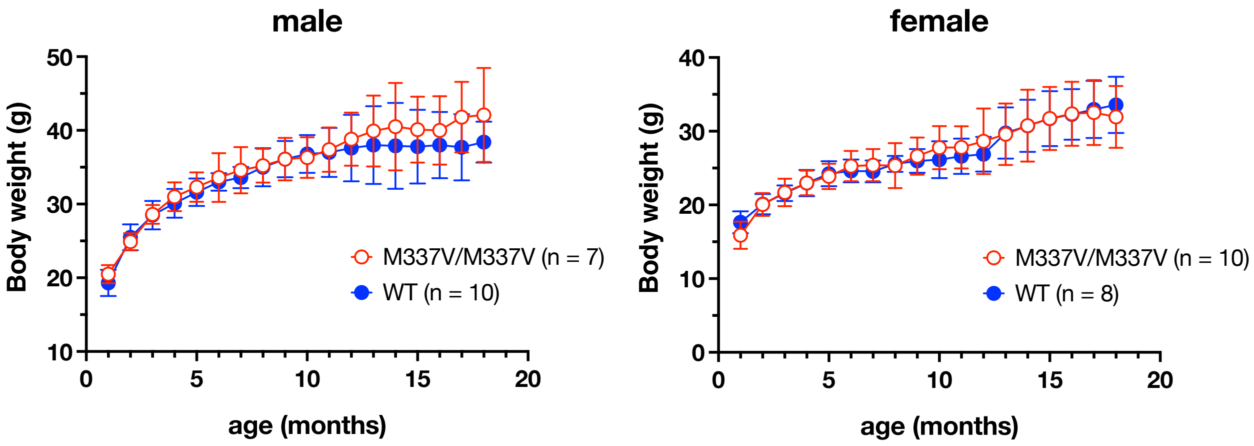

Figure S2. Body weights were not affected in TDP-43^{M337V} knock-in mice.

Body weights of male and female homozygous TDP-43^{M337V} knock-in (M337V/M337V) mice were measured at indicated ages and plotted as mean ± standard deviation (SD). No significant difference in the body weights among each genotype was observed.

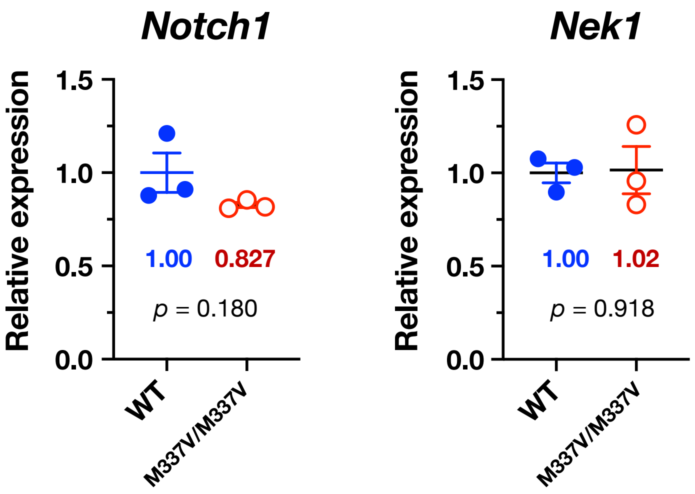

Figure S3. Relative expression levels of *Notch1* and *Nek1* mRNAs were not altered in brain of aged (700 days-old) homozygous TDP-43^{M337V} mice.

TDP-43^{M337V} mutation did not alter the mRNA levels of its representative downstream targets of TDP-43 reported in the previous studies including ours; *Notch1*[4] and *Nek1*[6]. Relative expression of mRNA levels normalized to the WT control are plotted with SD. n=3 each

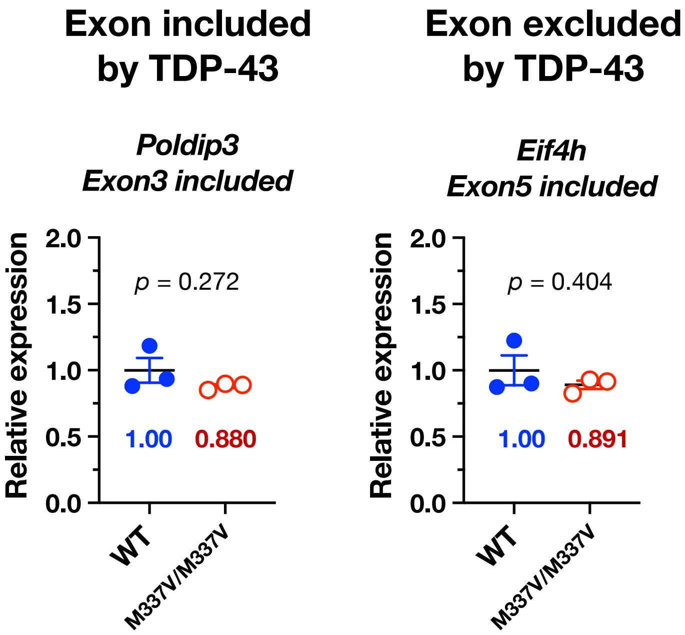

Figure S4. Splicing was not altered in *Eif4h* or *Poldip3*, which are also regulated by TDP-43, in brain of aged (700 days-old) homozygous TDP-43^{M337V} mice.

Relative expression of mRNA levels normalized to the WT control are plotted with SD. n=3 each.

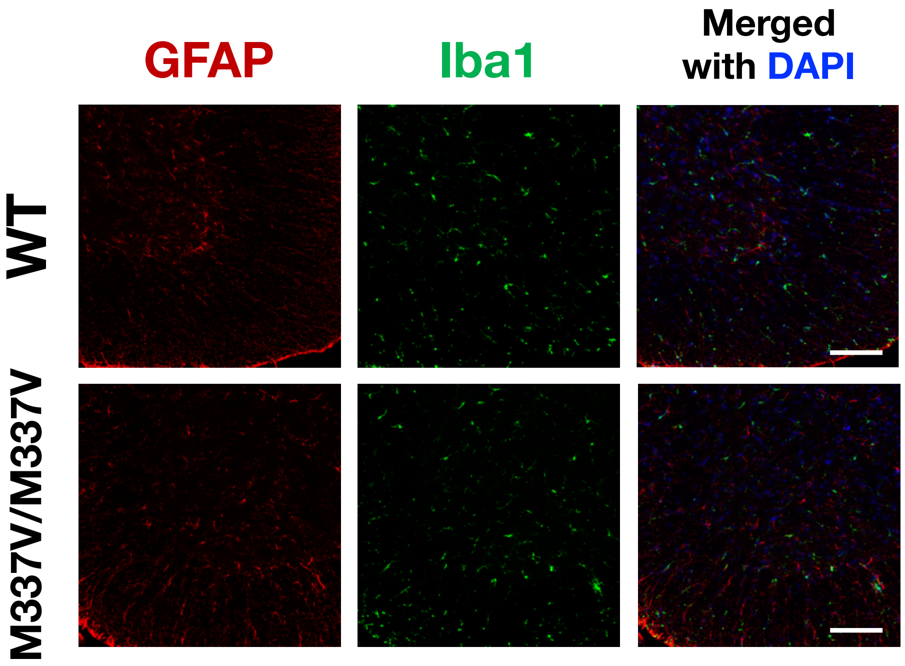

Figure S5. Gliosis was not observed in ventral horn of aged (700 days-old) homozygous TDP-43^{M337V} mice.

The spinal cord sections were immunostained with anti-GFAP, an astrocyte marker, and anti-Iba1, a microglia marker. Neither the increased number of the glial cells nor the morphological changes were observed. Scale bars = 200 µm.

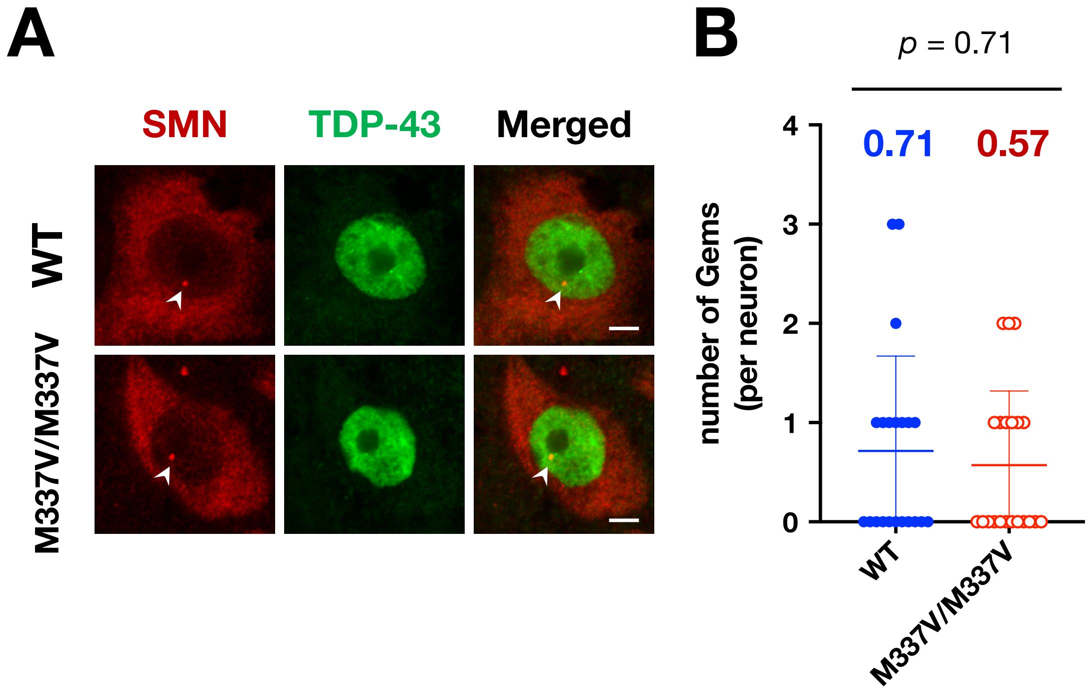

Figure S6. The number of Gems was not affected in ventral horn neurons of aged (700 days-old) homozygous TDP-43^{M337V} mice.

(A) Representative images of Gems in ventral horn neurons visualized with staining by anti-SMN and anti-TDP-43 antibodies. Nuclear foci with a concentration of SMN indicates the Gems (arrowheads). Scale bars = 5 µm. (B) The number of Gems in ventral horn neurons was not altered between WT control and TDP-43$^{M337V/M337V}$ mice. Data are plotted as mean with SD (n = 21 from two animals per each genotype).

3. Additional References

1. Aida T, Chiyo K, Usami T, Ishikubo H, Imahashi R, Wada Y, Tanaka KF, Sakuma T, Yamamoto T, Tanaka K: **Cloning-free CRISPR/Cas system facilitates functional cassette knock-in in mice.** *Genome Biol* 2015, **16:**87.
2. Singh P, Schimenti JC, Bolcun-Filas E: **A mouse geneticist's practical guide to CRISPR applications.** *Genetics* 2015, **199:**1-15.
3. Guyenet SJ, Furrer SA, Damian VM, Baughan TD, La Spada AR, Garden GA: **A simple composite phenotype scoring system for evaluating mouse models of cerebellar ataxia.** *J Vis Exp* 2010.
4. Nishino K, Watanabe S, Shijie J, Murata Y, Oiwa K, Komine O, Endo F, Tsuiji H, Abe

M, Sakimura K, et al: **Mice deficient in the C-terminal domain of TAR DNA-binding protein 43 develop age-dependent motor dysfunction associated with impaired Notch1-Akt signaling pathway.** *Acta Neuropathol Commun* 2019, **7:**118.
5. David FP, Rougemont J, Deplancke B: **GETPrime 2.0: gene- and transcript-specific qPCR primers for 13 species including polymorphisms.** *Nucleic Acids Res* 2017, **45:**D56-D60.
6. White MA, Kim E, Duffy A, Adalbert R, Phillips BU, Peters OM, Stephenson J, Yang S, Massenzio F, Lin Z, et al: **TDP-43 gains function due to perturbed autoregulation in a Tardbp knock-in mouse model of ALS-FTD.** *Nat Neurosci* 2018, **21:**552-563.
